# Coronary Heart Disease and Cardiovascular Risk Factors in Patients With Idiopathic Inflammatory Myopathies: A Systemic Review and Meta-Analysis

**DOI:** 10.3389/fmed.2021.808915

**Published:** 2022-01-14

**Authors:** Li Qin, Fang Li, Qiang Luo, Lifang Chen, Xiaoqian Yang, Han Wang

**Affiliations:** ^1^Department of Cardiology, The Affiliated Hospital of Southwest Jiaotong University, The Third People's Hospital of Chengdu, Chengdu, China; ^2^Department of Rheumatology, The Affiliated Hospital of Southwest Jiaotong University, The Third People's Hospital of Chengdu, Chengdu, China

**Keywords:** idiopathic inflammatory myopathies, coronary heart disease, hypertension, diabetes mellitus, dyslipidemia, meta-analysis

## Abstract

**Objectives:**

It is well-established that the association between atherosclerotic cardiovascular diseases (ASCVD) and connective tissue diseases (CTDs), but the relationship between coronary heart disease (CHD) and idiopathic inflammatory myopathies (IIMs) remains controversial yet. The aim of this meta-analysis is to systematically evaluate the risk of CHD in IIMs patients. In addition, we explore differences in traditional cardiovascular risk factors between IIMs patients and controls.

**Methods:**

We searched Pubmed, EMBASE and Cochrane databases to identify relevant observational studies published in English up to August 2021. Pooled relative risk (RR) and 95% confidence interval (CI) was calculated using the generic inverse variance method for the risk of CHD. A meta-proportion analysis was conducted to assess differences in cardiovascular risk factors between two groups.

**Results:**

A total of 15 studies met inclusion criteria: seven studies focused on CHD and nine studies focused on traditional cardiovascular risk factors. The results demonstrated that IIMs patients had a higher risk of CHD (RR = 2.19, 95% CI: 1.40–3.42). Hypertension (OR = 1.44, 95% CI: 1.28–1.61), diabetes mellitus (OR = 1.67, 95% CI: 1.55–1.81) and dyslipidemia (OR = 1.48, 95% CI: 1.19–1.84) were more prevalent in IIMs patients compared with controls. However, there was a significant heterogeneity among studies assessing the risk of CHD and hypertension. Subgroup analysis demonstrated that definition of CHD, country and sample size may be potential sources of heterogeneity.

**Conclusions:**

IIMs patients were at increased risk of CHD, and traditional cardiovascular risk factors appeared more prevalent in IIMs patients. This systemic review offers the proof that early appropriate interventions could reduce cardiovascular-associated morbidity and mortality in IIMs patients.

## Introduction

Idiopathic inflammatory myopathies (IIMs), also referred to generally as myositis, are a group of rare, chronic inflammatory autoimmune disorders, with the annual incidence and prevalence rate of 5.8–7.9 per 100,000 person-years, 14.0–17.4 per 100,000 person-years, respectively (1). IIMs mainly include dermatomyositis (DM), polymyositis (PM), inclusion body myositis, autoimmune necrotizing myopathy, overlap myositis and antisynthetase syndrome (2). DM and PM are the most common subtypes, which are characterized by inflammatory infiltration of the skeletal muscle and progressive proximal muscle weakness. IIMs often involve multiple organs, including the heart, lungs, kidney, skin, and gastrointestinal tract. More and more studies demonstrated that the risk of cardiovascular involvement was much higher in patients with IIMs than in the general population (3, 4). It is noted that cardiovascular disease (CVD) was the major cause of death in IIMs patients (3, 4). Previous studies reported that almost 10–20% of the deaths of patients can be attributed to cardiovascular events, such as coronary heart disease (CHD), heart failure (HF), complete heart block, and myocarditis (3, 4). In fact, CHD was not uncommon in IIMs patients, as it occurred in about 15.1 person-years of DM patients and 30.1 person-years of PM patients (5). However, the exact mechanisms of CHD in IIMs patients are still unclear, which need further exploration to strengthen medical aids. The increasing evidence revealed that chronic inflammatory and immune mechanisms may play an important role in the pathogenesis of CHD in patients with IIMs (6). In addition, traditional cardiovascular risk factors, such as hypertension, diabetes mellitus, dyslipidemia, smoking, obesity, or a sedentary lifestyle may also increase the risk of CHD in IIMs patients (3, 5). Based on the existing literature data, hypertension, diabetes mellitus and dyslipidemia are the most important risk factors among these factors (5–7). However, there is no systematic review to explore whether the three key traditional cardiovascular risk factors (hypertension, diabetes mellitus, and dyslipidemia) varied between IIMs patients and non-IIMs individuals.

It is worth mentioning that multiple studies have exhibited a strict association between connective tissue diseases (CTDs) and CHD risk, such as rheumatoid arthritis (RA), systemic lupus erythematosus (SLE), and systemic sclerosis (SSc), but the risk of CHD in IIMs patients is still inconclusive due to the conflicting epidemiological studies (8–10). Some studies demonstrated an increased risk of CHD among IIMs patients compared with the general population (11–16), but the study by Linos et al. found that there was no significant association between DM and CHD risk (17). So far, there is only one systemic review and meta-analysis to assess the risk of CHD in patients with IIMs, while it contains only four studies and is not enough to draw strong conclusions (18). Moreover, a number of studies focused on CHD risk among IIMs patients have been published after the study by Ungprasert et al. (11–13, 16). Thus, we perform a systemic review and meta-analysis of all published observational studies which compare the risk of CHD between IIMs patients and control subjects. Besides, we also perform a systemic review and meta-analysis of the three most important traditional cardiovascular risk factors (hypertension, diabetes mellitus, and dyslipidemia) in IIMs compared to the non-IIMs individuals.

## Materials and Methods

### Search Strategy

This systemic review and meta-analysis was conducted according to the recommendation of Meta-Analyses of Observational Studies in Epidemiology group (MOOSE) and the Preferred Reporting Items for Systematic Reviews and Meta-Analyses (PRISMA) statement (19, 20). Two investigators independently and systematically searched for eligible studies in three databases (Pubmed, EMBASE and Cochrane Library) from inception to August 2021. The following search terms were used in the search for studies on CHD risk: (“dermatomyositis” OR “polymyositis” OR “myositis” OR “idiopathic inflammatory myopathies”) AND (“coronary artery disease” OR “coronary heart disease” OR “coronary disease” OR “myocardial infarction” OR “coronary stenosis” OR “angina pectoris” OR “coronary thrombosis”). Besides, the following search terms were used in the search for studies on cardiovascular risk factors: (“dermatomyositis” OR “polymyositis” OR “myositis” OR “idiopathic inflammatory myopathies”) AND (“hypertension” OR “diabetes mellitus” OR “dyslipidemias” OR “hyperlipidemias”). The detailed search strategy was presented in the Supplementary Material. In addition, we also manually searched the references of retrieved articles to obtain more resources. Notably, only articles published in English were selected.

### Inclusion and Exclusion Criteria

The inclusion criteria of this review were as follows: (1) Although it is currently recommended to use the new EULAR/ACR classification criteria for adult and juvenile IIMs and their major subgroups (21), studies focused on the risk of CHD or traditional cardiovascular risk factors in IIMs patients are mostly published before the development of the new criteria. Moreover, the diagnosis of IIMs was mostly based on the criteria of Bohan and Peter in the previous studies (22). Therefore, patients included in the current review had to meet the criteria of Bohan and Peter in 1975 or the diagnostic code of the International Classification of Diseases (ICD), (2) all published observational studies (cohort studies and case-control studies) assessing the risk of CHD or considering traditional cardiovascular risk factors (hypertension, diabetes mellitus and dyslipidemia) in IIMs patients and controls, (3) odds ratios (ORs), relative risks (RRs), hazard ratios (HRs), standardized incidence ratios (SIRs), corresponding 95% confidence intervals (CIs), or enough original data to calculate the aforementioned parameters were provided, (4) non-IIMs subjects were identified as the control groups. The exclusion criteria were as follows: (1) conference abstracts, commentary, and the sample size of the study <20 in case or control group, (2) studies focused on specific population groups, such as juveniles (<18 years) or pregnant IIMs patients, (3) publications from the same database (in which case the study reporting the most comprehensive results was selected). Each investigator determined the study eligibility independently, and any disagreements were resolved by consulting the senior investigator.

### Data Extraction and Quality Assessment

Relevant data of the eligible studies were independently collected by two reviewers. The following information was extracted from each study: last name of the first author, year of publication, country, study design, sample size, demographic characteristics of the study population, and study results. The quality of included studies was assessed according to the Newcastle-Ottawa Scale (NOS), which mainly included three domains (study selection, comparability, and exposure/outcome) with eight items (23). The higher the total score, the better the study quality. In general, a study with a total score of 6 and above was considered to be of high quality. If encountered with inconsistent results, they were settled through consensus.

### Definition of End Points

Our primary outcomes of interest were CHD, hypertension, diabetes mellitus and dyslipidemia, and these end points were treated as dichotomous variables. CHD included angina pectoris, myocardial infraction (MI) and unstable angina, which diagnosis was based on patients' medical charts, registry databases, or self-reports. Hypertension was defined as blood pressure consistently above 140/90 mmHg, or antihypertensive drugs use, or diagnosis of hypertension in medical charts, registry databases, or self-reports. Diabetes mellitus was defined as fasting blood glucose ≥7.0 mmol/L, or typical symptoms of diabetes mellitus with random blood glucose ≥11.1 mmol/L, or glycosylated hemoglobin (HbA1c) ≥6.5%, or intake of hypoglycemic medications, or diagnosis of diabetes in medical records, registry databases, or self-reports. Dyslipidemia was defined as triglyceride (TG) level ≥150 mg/dL, total cholesterol (TC) level ≥220 mg/dL, low-density lipoprotein cholesterol (LDL-C) level ≥140 mg/dL, high-density lipoprotein cholesterol (HDL-C) level ≤ 40 mg/dL, or use of lipid-lowering medications, or diagnosis of dyslipidemia in medical charts, registry databases, or self-reports.

### Statistical Analysis

All statistical analyses were performed using Review Manager 5.4 software (The Cochrane Collaboration, Oxford, UK) and Stata 16.0 software (Stata Corporation, College Station, TX, USA). As the outcome of interest (CHD risk) was relatively uncommon, and only one case-control study reported the estimate as OR, thus we use OR as an estimate for RR to calculate the pooled effect estimates. Moreover, adjusted point estimates and standard errors were collected from individual studies and were combined by using the generic inverse variance method of DerSimonian and Laird (24). The meta-proportion analysis was conducted to assess differences in cardiovascular risk factors between IIMs patients and controls. The Cochran's Q test and *I*^2^ statistics were applied to evaluate statistical heterogeneity among studies, and a higher *I*^2^ value indicating a higher level of heterogeneity. An *I*^2^ value of 0–25, 25–50, 50–75, and 75–100%, was considered as insignificant, low, moderate, and high heterogeneity, respectively (25). The fixed-effect model was employed when *P* > 0.1 for the Q test and *I*^2^ < 50% for the *I*^2^ test; otherwise, the random-effect model was adopted. When heterogeneity was significant (*I*^2^ > 50%), subgroup analysis or sensitivity analysis was conducted to explore the potential source of heterogeneity. In the current study, funnel plot analysis could not be conducted as <10 studies were included in each meta-analysis. All tests were two-tailed, and *P* < 0.05 was considered to be statistically significant except for assessing the presence of heterogeneity.

## Results

**Figure 1:**
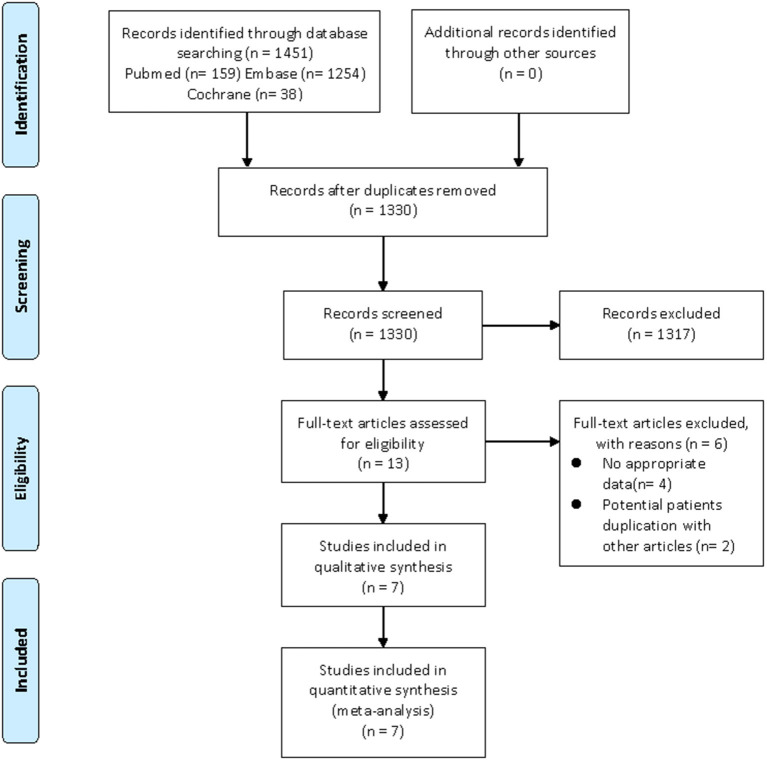
Flow diagram of the selection process for studies on CHD risk. From Moher et al. (20).

**Figure 2:**
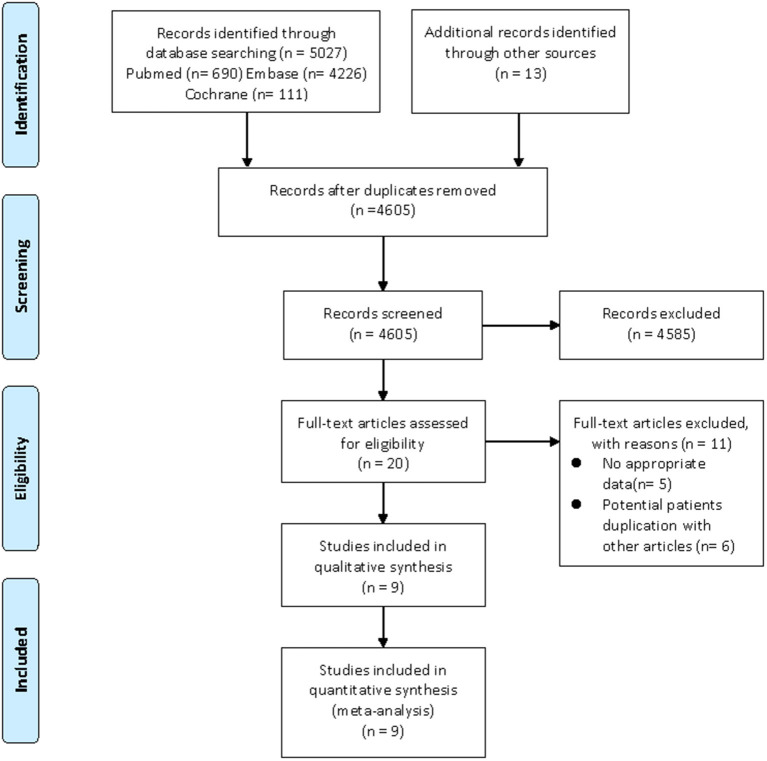
Flow diagram of the selection process for studies on cardiovascular risk factors. From Moher et al. (20).

Our search strategy yielded a total of 1,451 and 5,040 potentially relevant articles associated with CHD risk and cardiovascular risk factors, respectively. After eliminating duplicate articles, the remaining 1,330 and 4,605 articles were screened based on titles and abstracts, respectively. At this stage, 1,317 and 4,585 studies were excluded as they were unrelated to the current study, and the remaining 13 and 20 articles were subjected to full-text review. Of them, nine articles (CHD, four articles; cardiovascular risk factors, five articles) were excluded due to lacking available data, and 8 articles (CHD, two articles; cardiovascular risk factors, six articles) were also excluded as they utilized the same database. For example, the study by Antovic et al. (26) and the study by Moshtaghi-Svensson et al. (27) used the same databases (the Swedish National Patient Register Database and the Swedish Population Register Database), but the latter reported the most comprehensive results, thus, we only included the study by Moshtaghi-Svensson et al. in the current review. Finally, seven articles focused on CHD risk and nine articles focused on cardiovascular risk factors met the inclusion criteria and were included in this meta-analysis (11–17, 27–34). Moreover, the majority of eligible studies in the current review were about patients with DM/PM, but these studies did not describe the different subtypes of PM or DM in detail. It was noteworthy that some studies separately listed the outcomes of interest in patients with PM and DM, so we also described them separately in the meta-analysis. The flow chart of the literature screening was shown in Figures 1, 2.

### Study Characteristics and Quality Assessment

In all eligible studies, seven studies (six cohort studies and one case-control study) were relevant to CHD risk and nine studies (five cohort studies and four case-control studies) were relevant to traditional cardiovascular risk factors. Among the studies on the risk of CHD, the number of IIMs patients ranged from 350 to 10,156, with the mean age of IIMs patients ranging from 46.1 to 62.4 years, and the proportion of females in the case group ranged between 45 and 73.2%. Moreover, five of these studies strictly defined CHD as MI or acute coronary syndrome (ACS) (11–14, 16), while the remaining two studies used broader definitions of CHD (15, 17). The study by Linos et al. (17) defined CHD as AMI, angina, and coronary intervention (coronary artery bypass grafting or percutaneous transluminal coronary angioplasty), but in another study, CHD was defined as AMI, angina and chronic CHD (15). Among the studies on traditional cardiovascular risk factors, the number of IIMs patients ranged from 28 to 4958, with the mean age of IIMs patients ranging from 42.9 to 62.2 years, and the ratio of females in case group ranged between 56 and 78.6%. Only two studies fully documented three traditional cardiovascular risk factors (30, 31), while the majority of studies only contained one or two cardiovascular risk factors. Furthermore, the included studies were conducted in many countries, including China (11, 33, 34), Canada (13, 14, 29), Sweden (15, 16, 27), United States (US) (17, 28), United Kingdom (UK) (12, 30), Italy (31), and Australia (32). Besides, the control group was matched according to age and gender with the case group in each study. In addition, all studies in this meta-analysis were journal articles with high quality (NOS score ≥6), with an average NOS score of 6.87. The detailed characteristics and quality assessment of these studies were illustrated in Tables 1, 2.

**Table 1:** Main characteristics of the included studies on CHD in this meta-analysis.

**References**	**Country**	**Study design**	**Patients**	**Controls**	**CHD**	**Follow up**	**Average range of follow-up**	**Number of T/C**	**Mean age of T/C, years**	**Female (T/C), %**	**Confounders adjusted**	**Effect estimate (95% CI)**	**Quality score (NOS)**
Leclair et al. (16)	Sweden	Cohort study	All patients who were diagnosis with IIMs between 2002 and 2011. Cases were identified by using the Swedish National Patient Register indexes data.	Age-, gender-, and residential area-matched subjects randomly selected from the same database.	ACS	Until the first of ACS, first emigration, death or 31 December 2013	IIMs: 4.5 years; Controls: 6.0 years	655/6,831	60/61	56/56	Age, gender, residential area	HR = 2.4 (1.8–3.2)	4/1/2
Lin et al. (11)	China	Cohort study	All patients who were diagnosed with PM or DM between 1998 and 2010. Cases were identified by using the National Health Insurance Research Database.	Age-, sex- and entry time-matched general population selected from the same database.	ACS	Until ACS diagnosis or censored for loss of follow-up, death, withdrawal from the insurance programme, or the end of 2010	10 years	2,029/8,116	46.1/45.7	67.8/67.8	Age, sex, hypertension, diabetes, hyperlipidemia, cerebrovascular accident, and chronic obstructive pulmonary disease	HR = 1.98 (1.17–3.35)	4/1/3
Linos et al. (17)	US	Case-control study	All hospitalized patients who were diagnosis of DM between 1993 and 2007. Cases were identified by using the Healthcare Cost and Utilization Project Nationwide Inpatient Sample database.	Age- and gender-matched subjects randomly selected from the same database.	AMI, angina, coronary intervention	NA	NA	10,156/76,440	58.3/58.5	73.2/73.4	Age, gender	OR = 0.96 (0.9 1–1.01)	3/1/2
Párraga Prieto et al. (12)	UK	Cohort study	All patients who were diagnosed with PM or DM between 1987 and 2013. Cases were identified by using the UK Clinical Practice Research Datalink.	Age- and gender-matched healthy subjects randomly selected from the same database.	MI	Until occurrence of any fatal and non-fatal major cardiovascular events	7 years	603/4,061	58/52	64/63	Age, gender, diabetes, hypertension, smoking status	HR = 1.61 (1.27–2.04)	4/1/2
Rai et al. (13)	Canada	Cohort study	All patients who were diagnosed with PM or DM between 1 January 1996 and 31 December 2010. Cases were identified by using Population Data British Columbia.	Age-, sex- and entry time matched general population selected from the same database.	MI	Until occurrence of MI, stroke, died, dis-enrolled from the health plan, or 31 December 2010	NA	(1) PM: 424/4,426, (2) DM: 350/3,497	(1) PM: 60/NA, (2) DM: 56/NA	(1) PM: 59/NA, (2) DM: 65/NA	(1) PM: age, sex, entry-time, number of outpatient visits, glucocorticoids and angina; (2) DM: age, sex, entry-time, number of outpatient visits, NSAIDs and cardiovascular drugs	(1) PM: HR = 3.89 (2.28–6.65), (2) DM: HR = 2.92 (1.48–5.78)	4/2/2
Tisseverasinghe et al. (14)	Canada	Cohort study	All patients who were diagnosed with PM or DM between 1994 and 2003. Cases were identified by using the Quebec provincial database.	Using Canadina age- and sex-matched general population incidence rates for AMI as the comparator for the calculation of standardized incidence ratio.	AMI	Until the first of outcome event, death, or 31 December 2003	4 years	607/NA	62.4/NA	70/NA	Age, sex	RR = 1.95 (1.40–2.73)	4/1/2
Zöller et al. (15)	Sweden	Cohort study	All patients who were hospitalized with a main diagnosis of PM or DM between 1 January 1964 and 31 December 2008. Cases were identified by using the Swedish national data registers.	Using Swedish age- and sex-specific general population incidence rates of AMI, angina, and chronic coronary heart disease as the comparator for the calculation of standardized incidence ratio.	AMI, angina, chronic CHD	Until hospitalization for CHD, death, emigration, or 31 December 2008	NA	1,531/NA	NA	45/NA	Age, sex	RR = 3.82 (2.68–5.44)	4/1/2

*CHD, coronary heart disease; T/C, patients/controls; NOS, Newcastle-Ottawa Scale; ACS, acute coronary syndrome; HR, hazard ratio; PM, dermatomyositis; DM, polymyositis; US, United States; AMI, acute myocardial infarction; NA, not applicable; OR, odds ratio; UK, United Kingdom; MI, myocardial infarction; NSAIDs, non-steroidal anti-inflammatory drugs; RR, risk ratio*.

**Table 2:** Main characteristics of the included studies on cardiovascular risk factors in this meta-analysis.

**References**	**Country**	**Study design**	**Patients**	**Controls**	**Number of T/C**	**Mean age of T/C, years**	**Female (T/C), %**	**Number of hypertension (T/C)**	**Number of diabetes mellitus (T/C)**	**Number of dyslipidemia (T/C)**	**Quality score (NOS)**
Bae et al. (28)	US	Case-control study	All patients diagnosed with IIMs were verified by chart review. Cases were recruited from the University of California, Los Angeles.	Age- and sex-matched healthy subjects selected from the same database.	95/41	NA/49	72.6/68.3	28/8	16/1	NA	3/1/2
Carruthers et al. (29)	Canada	Cohort study	All patients who were diagnosed with PM or DM between January 1996 and December 2010. Cases were identified by using the Population Data British Columbia.	Age-, sex-and calendar year of study entry-matched general population randomly selected from the same database.	(1) PM: 443/4,603, (2) DM: 355/3,577	(1) PM: 60.39/60.53, (2) DM: 55.9/55.8	(1) PM: 58.0/58.2, (2) DM: 64.5/64.5	(1) PM: 136/1,222, (2) DM: 96/772	NA	NA	4/2/2
D'Silva et al. (30)	UK	Cohort study	All patients who were diagnosed with PM or DM between 1996 and 2014. Cases were identified by using the Health Improvement Network database.	Age-, sex-and database entry year-matched subjects selected from the same database.	(1) PM: 407/3,648, (2) DM: 410/3,763	(1) PM: 59/59, (2) DM: 57.5/57.5	(1) PM: 60.7/60.7, (2) DM: 65.6/65.9	(1) PM: 133/1,026, (2) DM: 127/945	(1) PM: 35/256, (2) DM: 32/233	(1) PM: 50/295, (2) DM: 41/306	4/1/2
Guerra et al. (31)	Italy	Case-control study	All patients who were diagnosis with PM or DM between April 2015 and June 2016. Cases were identified by using the consecutively referred to the Clinical Medical.	Age-, sex-and cardiovascular risk factors-matched subjects selected from the out-of-hospital Cardiology Clinic.	28/28	61.3/63.6	78.6/78.6	12/12	2/2	4/4	3/2/2
Khoo et al. (32)	Australia	Case-control study	All patients who were diagnosis with IIMs between 1995 and 2014. Cases were identified by using the South Australian Myositis Database.	Age- and gender-matched general population selected from The North West Adelaide Health Study cohort.	221/662	62.2/62.1	59.7/59.7	NA	9/11	NA	3/1/2
Párraga Prieto et al. (12)	UK	Cohort study	All patients who were diagnosed with PM or DM between 1987 and 2013. Cases were identified by using the UK Clinical Practice Research Datalink.	Age- and gender-matched healthy subjects randomly selected from the same database.	603/4,061	58/52	64/63	253/1,352	132/600	NA	4/1/2
Moshtaghi-Svensson et al. (27)	Sweden	Cohort study	All patients who were diagnosis with IIM between 2002 and 2011. Cases were identified by using the Swedish National Patient Register indexes data.	Age-, gender-, and residential area-matched general population randomly selected from the Total Population Register database.	663/6,673	61/61	56/56	98/580	35/256	NA	3/1/2
Wang et al. (33)	China	Case-control study	All patients who were diagnosed with PM between September 2009 and February 2013. Cases were recruited from the cardiology department of the No.3 Hospital of Chengdu and the rheumatology department of West China Hospital.	Age- and sex-matched healthy subjects.	60/60	42.9/42.9	73.3/73.3	NA	NA	31/17	3/1/2
Wu et al. (34)	China	Cohort study	All patients who were diagnosis with DM between 1 January 1998 and 31 December 2007. Cases were identified by using the Registry of Catastrophic Illness Database.	Age-, sex-and index data-matched subjects selected from the Longitudinal Health Insurance Database.	4958/19,832	52.89/52.89	59.9/59.9	1,358/3,666	711/1,731	NA	3/1/3

*US, United States; T/C, patients/controls; NOS, Newcastle-Ottawa Scale; NA, not applicable; PM, dermatomyositis; DM, polymyositis; UK, United Kingdom; IIMs, idiopathic inflammatory myopathies*.

### Meta-Analysis Results

#### CHD

**Figure 3:**
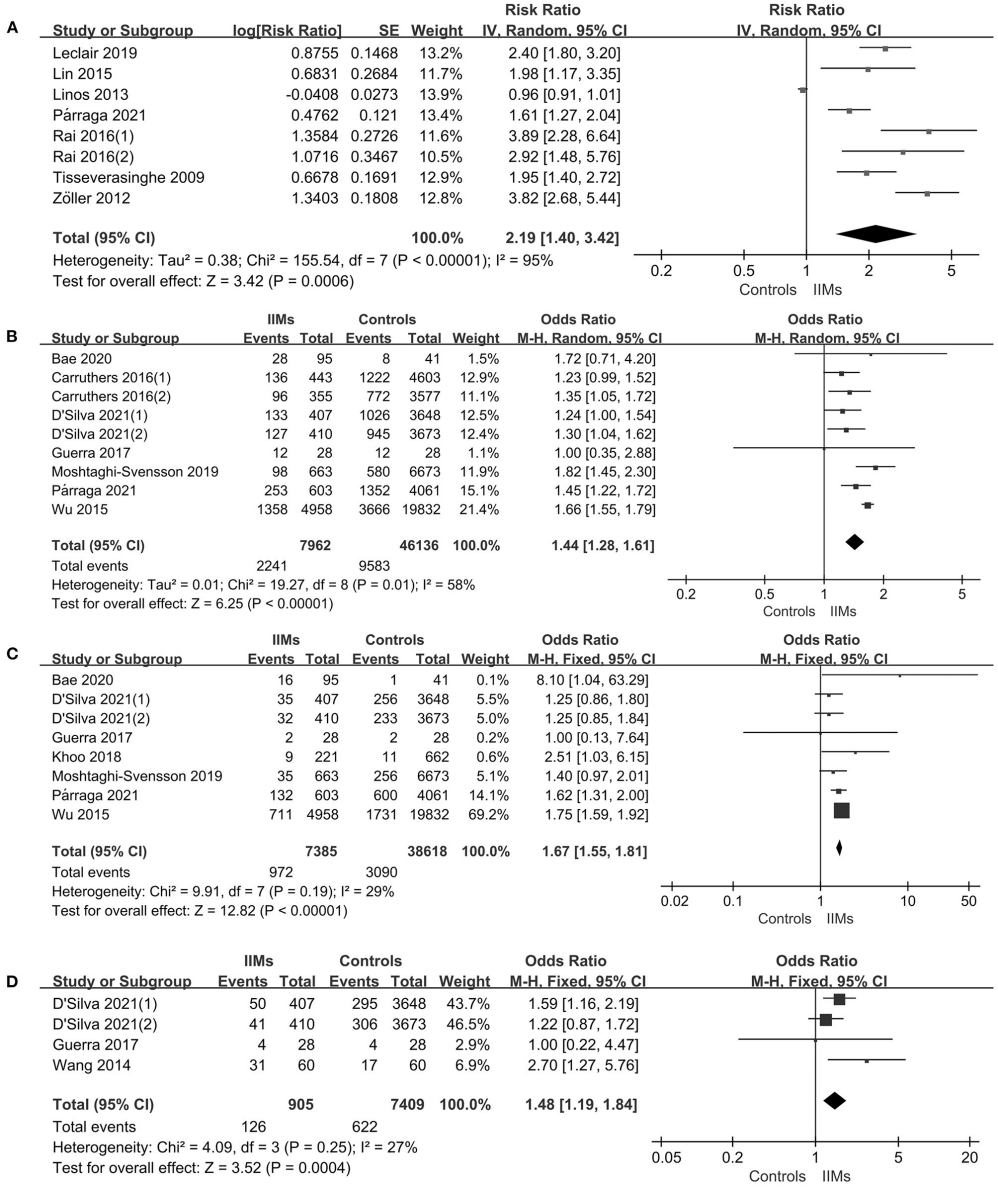
**(A)** Comparison of CHD risk between IIMs patients and controls. **(B)** Comparison the prevalence of hypertension between IIMs patients and controls. **(C)** Comparison of the prevalence of diabetes mellitus risk between IIMs patients and controls. **(D)** Comparison the prevalence of dyslipidemia risk between IIMs patients and controls. IIMs, idiopathic inflammatory myopathies; CHD, coronary heart disease.

The forest plots of the meta-analysis were presented in Figure 3A. The results demonstrated a statistically significant increased CHD risk in IIMs patients with the pooled risk ratio of 2.19 (95% CI: 1.40–3.42, *P* = 0.0006), but the heterogeneity was high among the studies (*I*^2^ = 95%, *P* < 0.00001). Therefore, we conducted the subgroup analysis and sensitivity analysis. Subgroup analysis categorized by the country and the definition of CHD, and the results were summarized in Table 3. The results showed that patients with IIMs had a higher risk of CHD in Canada (RR = 2.68, 95% CI: 1.70–4.23) and Sweden (RR = 2.99, 95% CI: 1.90–4.72), but this finding was not suitable for studies from other countries, including China, US and UK. The heterogeneity of Canada and Sweden subgroups was relatively low, with *I*^2^ statistics of 60 and 75%, respectively. What's more, the pooled RR of subgroup in MI and ACS was 2.27 and 2.30, indicating that MI and ACS were indeed more prevalent in patients with IIMs compared with non-IIMs population. Besides, sensitivity analysis was conducted by sequentially omitting each individual study to assess the stability of the results. The pooled estimates did not significantly change at each step, suggesting that the results of this meta-analysis were relatively robust. Notably, all cohort studies presented that there was an increased risk of CHD in IIMs patients, but the only case-control study did not show an increased risk. When excluding this case-control study, the pooled RR of all cohort studies was 2.44 (95% CI: 1.86–3.21), with a relatively low heterogeneity (*I*^2^ = 73%).

**Table 3:** Subgroup analysis of CHD risk in IIMs patients.

**Subgroups**		***N***	**RR (95% CI)**	***Z***	***P***	**Heterogeneity test**		
						***Q***	***I*^2^, %**	***P***
Country	Canada	2	2.68 (1.70–4.23)	4.25	<0.0001	4.95	60	0.08
	Sweden	2	2.99 (1.90–4.72)	4.72	<0.00001	3.98	75	0.05
	Others	3	1.39 (0.88–2.20)	1.40	0.16	24.09	92	<0.00001
	Combined	7	2.19 (1.40–3.42)	3.42	0.0006	155.54	95	<0.00001
Definition of CHD	MI	3	2.27 (1.56–3.30)	4.31	<0.0001	10.28	71	0.02
	ACS	2	2.30 (1.78–2.96)	6.45	<0.00001	0.40	0	0.53
	Others	2	1.89 (0.49–7.33)	0.92	0.36	57.05	98	<0.00001
	Combined	7	2.19 (1.40–3.42)	3.42	0.0006	155.54	95	<0.00001

*CHD, coronary heart disease; IIMs, idiopathic inflammatory myopathies; RR, risk ratio; MI, myocardial infarction; ACS, acute coronary syndrome*.

#### Hypertension

As shown in Figure 3B, there were seven studies describing the prevalence of hypertension in a total of 7,962 IIMs patients and 46,136 controls (12, 27–31, 34). The results revealed that there was a 44% increase in the prevalence of hypertension in IIMs patients vs. control subjects (OR = 1.44, 95% CI: 1.28–1.61, *P* < 0.00001), but the statistical heterogeneity was significant among these studies, with an *I*^2^ of 58% (*P* = 0.01). Notably, these seven studies were conducted in six different countries, and the number of IIMs patients ranged from 28 to 4,958 among these studies. We assumed that the different geographic regions of the country and sample size may be the potential source of heterogeneity, so we performed a subgroup analysis according to the above variables. In our study, a large sample study was defined as the number of case groups >500, otherwise, the study was categorized as a small sample study. The results of our subgroup analysis were listed in Table 4. In terms of geographic regions, IIMs patients in Europe, North America and Asia all had a significantly increased frequency of hypertension than controls. With regard to sample size, the subgroup analysis also demonstrated that IIMs patients had a higher prevalence of hypertension compared with the controls in both large sample study (OR = 1.63, 95% CI: 1.48–1.80) and small sample study (OR = 1.24, 95% CI: 1.14–1.42). The sensitivity analysis indicated that no single study had a significant influence on the pooled OR, suggesting that the findings were stable and reliable. What's more, there was also no significant change in the pooled OR of the remaining cohort study (OR = 1.44, 95% CI: 1.27–1.62) after excluding only two case-control studies, but statistical heterogeneity still existed among the cohort studies (*I*^2^ = 68%).

**Table 4:** Subgroup analysis of the prevalence of hypertension in IIMs patients.

**Subgroups**		***N***	**OR (95% CI)**	***Z***	***P***	**Heterogeneity test**		
						***Q***	***I*^2^, %**	***P***
Geographic areas	Europe	4	1.42 (1.23–1.65)	4.68	<0.00001	7.08	44	0.13
	North America	2	1.29 (1.10–1.51)	3.13	0.002	0.75	0	0.69
	Asia	1	1.66 (1.55–1.79)	13.86	<0.00001	NA	NA	NA
	Combined	7	1.44 (1.28–1.61)	6.25	<0.00001	19.27	58	0.01
Sample size	Small sample (<500)	4	1.24 (1.14–1.42)	4.29	<0.0001	1.04	0	0.96
	Large sample (≥500)	3	1.63 (1.48–1.80)	9.83	<0.00001	2.89	31	0.24
	Combined	7	1.44 (1.28–1.61)	6.25	<0.00001	19.27	58	0.01

*IIMs, idiopathic inflammatory myopathies; OR, odds ratio; NA, not applicable*.

#### Diabetes Mellitus

We identified seven studies with 7,385 IIMs patients that assessed the prevalence of diabetes mellitus (12, 27, 28, 30–32, 34). Overall, there was an increased prevalence of diabetes mellitus in patients with IIMs compared with the matched non-IIMs participants (OR = 1.67, 95% CI: 1.55–1.81, *P* < 0.00001), as presented in Figure 3C. Heterogeneity was not significant between studies (*I*^2^ = 29%, *P* = 0.19).

#### Dyslipidemia

A total of three studies including 905 IIMs patients and 7,409 controls that evaluated the prevalence of dyslipidemia in patients with IIMs (30, 31, 33), with an overall OR of 1.48 (95% CI: 1.19–1.84, *P* = 0.0004), implying that dyslipidemia was more prevalent in IIMs patients (Figure 3D). What's more, no significant heterogeneity was found among the studies (*I*^2^ = 27%, *P* = 0.25).

## Discussion

To our knowledge, the current study included the largest study population and provided the latest meta-analysis of the risk of CHD and the prevalence of cardiovascular risk factors (hypertension, diabetes mellitus and dyslipidemia) in IIMs patients. The results of our study demonstrated an increased risk of CHD in patients with IIMs in comparison with the non-IIMs subjects. Meanwhile, cardiovascular risk factors were more prevalent in IIMs patients. There was a significant heterogeneity among studies assessing the risk of CHD and the prevalence of hypertension.

This meta-analysis revealed a significant association between IIMs patients and CHD risk, and the risk of CHD in IIMs patients was 2.19 times higher than that in non-IIMs subjects, which was consistent with the results of the previous studies (11, 13, 18, 35). Although extreme heterogeneity existed in these included studies, the sensitivity analysis pointed out that the combined results of this meta-analysis were reliable. After subgroup analysis by country, the *I*^2^ statistics among Canada and Sweden subgroups had significant changes. However, the heterogeneity was still high in other countries subgroups, and the possible reasons were as follows. Firstly, these three studies were conducted in three different countries. Indeed, people living in different countries may have different life and eating habits, genetic and environmental characteristics, all of which may affect the risk of CHD. Nevertheless, there was only one article assessing the risk of CHD in these counties, so we were not able to draw a clear conclusion for these regions. Secondly, the sample sizes of the case group changed greatly, ranging from 655 to 10,156. Thirdly, the definitions of CHD and population selection of these studies were inconsistent. Two population-based studies defined CHD as ACS, while another hospital-based study used a broad definition. Besides, our subgroup analysis also showed a higher risk of MI and ACS in IIMs patients, which was similar to the results of previous studies (11–14, 16). Moreover, the heterogeneity of ACS subgroups had a remarkable reduction (*I*^2^ = 0%), thus we speculated that the definition of CHD may be the main source of heterogeneity.

This meta-analysis also indicated that IIMs patients were 1.44, 1.67, and 1.48 times more likely to be complicated with hypertension, diabetes mellitus, and dyslipidemia compared with non-IIMs individuals, respectively. However, significant heterogeneity among studies on hypertension in IIMs patients was present. We performed subgroup analysis stratified by geographic regions of the country and sample size of case group. The results indicated that the prevalence of hypertension in IIMs patients was greatly higher than that in the control group of all subgroups, with lower statistical heterogeneity. In addition, sensitivity analyses suggested that the results were convincing and robust, but the heterogeneity among cohort studies was still relatively higher. In order to investigate the source of heterogeneity among the cohort studies, we conducted further subgroup analysis based on sample size. The findings demonstrated that there was no obvious change in the pooled OR between the total and the two subgroups, but the heterogeneity among the large sample studies and small sample studies decreased significantly; *I*^2^ value reduced to 0 and 13%, respectively. Based on these results, we think that geographic regions of the country and sample size may be the main source of heterogeneity among the studies focused on hypertension. In the future, more prospective multicenter studies with large sample sizes are required to be conducted to confirm these results.

To date, the underlying etiology and pathogenesis of CHD in patients with IIMs are not completely known, but the increasing evidence suggests that inflammatory and immunological mechanisms may play a crucial role (14, 17, 35). Coronary atherosclerosis is the fundamental pathophysiological process of CHD. The relationship between inflammation and accelerated atherosclerosis has been well-described as inflammatory cytokines, oxidative stress and endothelial cell activation could contribute to endothelial damage and dysfunction, eventually resulting in atherosclerosis (36, 37). Moreover, systemic inflammation linked to autoimmune disease can also promote hypercoagulable state, which is another vital predisposing risk factor for the development of CHD (38). Besides, the previous study pointed out that there were autoimmune cell imbalance and immune dysregulation in patients with CHD (39). Abnormal proliferation of B lymphocytes can differentiate into plasma cells and produce higher levels of autoantibodies, especially myositis specific autoantibodies (MSAs) and myositis associated autoantibodies (MAAs), which not only can be used to classify IIMs subtypes, but also to identify IIMs clinical phenotypes (40, 41). A multicenter cohort study of IIMs patients found a significant association between anti-signal recognition particle (SRP) antibody and cardiac involvement (OR = 4.15, *P* = 0.004) (42). In addition, traditional cardiovascular risk factors, such as hypertension, diabetes mellitus and dyslipidemia, were more prevalent in IIMs patients compared with the general population, and they may influence the progression of atherosclerosis by triggering the inflammatory response. Furthermore, many studies showed that traditional cardiovascular risk factors and therapeutic drugs of IIMs such as glucocorticoids (GC) were also associated with the higher risk of CHD in patients with IIMs (14, 43, 44). For example, the study by Tisseverasingh et al. (14) demonstrated that there was a significant correlation between the incidence of acute MI and hypertension (RR = 2.6, 95% CI: 1.2–5.5) as well as dyslipidemia (RR = 2.6, 95% CI: 1.0–6.5).

It is worth noting that it remains questionable whether the high prevalence of cardiovascular risk factors can be attributed to IIMs itself or pharmacotherapy. IIMs are long-term and chronic autoimmune diseases in nature that require long-term treatment with medication, and GC are considered as first-line therapy in patients with IIMs (43, 44). Numerous studies proposed that long-term use of GC can bring cardiovascular adverse effects, such as increased the risk of cardiovascular risk factors (43, 44). Nevertheless, our previous studies revealed that a significantly increased frequency of dyslipidemia in untreated patients with PM or DM compared with age- and sex-matched healthy controls (33, 45). In addition, the study by Limaye et al. demonstrated that the prevalence of hypertension (62 vs. 9.4%) and diabetes mellitus (29 vs. 4%) was also higher in IIMs patients than that in the general population (46). However, there was no increase in the prevalence of hypertension and diabetes mellitus post-IIMs diagnosis compared to pre-IIMs diagnosis, which likely reflected that these cardiovascular risk factors did not present as a complication of treatment (46). Therefore, we speculated that IIMs itself may also be associated with an increased risk of cardiovascular risk factors in IIMs patients.

Even though this meta-analysis included relatively high-quality studies, there were still some limitations. First, considerable statistical heterogeneity was observed among the studies on CHD and hypertension, thus interpretation of the results should be cautious. Second, the majority of included studies were performed using medical registry-based databases, which cannot completely guarantee the accuracy of the data. Third, as the included studies were all observational studies, it was difficult to draw a causal relationship. Therefore, it was not clear whether IIMs itself or other potential confounding factors work together to increase the risk of CHD or the prevalence of traditional cardiovascular risk factors. Fourth, a subgroup analysis evaluating all the different subtypes of PM or DM that cannot be performed, which may limit the power of the analysis. In addition, due to few studies, we cannot assess the publication bias, but there may be some inevitable publication biases, as only published English studies were used. Overall, further prospective studies with larger sample sizes should be carried out to verify our findings.

## Conclusion

In conclusion, this meta-analysis revealed an increased risk of CHD in patients with IIMs compared with age- and gender-matched non-IIMs subjects. Meanwhile, traditional cardiovascular risk factors, such as hypertension, diabetes mellitus, and dyslipidemia, were more prevalent in IIMs patients. Besides, chronic inflammation, traditional cardiovascular risk factors and some therapeutic drugs of IIMs were associated with the increased risk of CHD. Therefore, clinicians ought to realize those associations, and adopt appropriate measures to reduce the risk of CVD in patients with IIMs.

## Data Availability Statement

The original contributions presented in the study are included in the article/Supplementary Material, further inquiries can be directed to the corresponding author/s.

## Author Contributions

LQ and HW: conception and design of the study. QL and XY: literature search and data collection. FL and LC: data analysis and interpretation. LQ, FL, and HW: supervision. All authors contributed to the article and approved the submitted version.

## Funding

This study was supported by the National Nature Science Foundation (grant number 81300243).

## Conflict of Interest

The authors declare that the research was conducted in the absence of any commercial or financial relationships that could be construed as a potential conflict of interest.

## Publisher's Note

All claims expressed in this article are solely those of the authors and do not necessarily represent those of their affiliated organizations, or those of the publisher, the editors and the reviewers. Any product that may be evaluated in this article, or claim that may be made by its manufacturer, is not guaranteed or endorsed by the publisher.
