# Supplementary material for: Coronary Heart Disease and Cardiovascular Risk Factors in Patients With Idiopathic Inflammatory Myopathies: A Systemic Review and Meta-Analysis
Source: Front Med (Lausanne). 2022 Jan 14;8:808915. doi: 10.3389/fmed.2021.808915 (PMC8795615; doi:10.3389/fmed.2021.808915)
Supplement: Supplementary file 1 [file Data_Sheet_1.PDF]

Search Strategy

1. Coronary heart disease

Database: Pubmed

1. "dermatomyositis"[MeSH]
2. "polymyositis"[MeSH]
3. "myositis"[MeSH]
4. "dermatomyositis"[Title/Abstract]
5. "polymyositis"[Title/Abstract]
6. "myositis"[Title/Abstract]
7. "idiopathic inflammatory myopathies"[Title/Abstract]
8. "idiopathic inflammatory myopathy"[Title/Abstract]
9. 1 OR 2 OR 3 OR 4 OR 5 OR 6 OR 7 OR 8
10. "coronary disease"[MeSH]
11. "coronary artery disease"[MeSH]
12. "coronary stenosis"[MeSH]
13. "myocardial infarction"[MeSH]
14. "coronary thrombosis"[MeSH]
15. "angina pectoris"[MeSH]
16. "coronary disease"[Title/Abstract]
17. "coronary diseases"[Title/Abstract]
18. "coronary artery disease"[Title/Abstract]
19. "coronary artery diseases"[Title/Abstract]
20. "coronary heart disease"[Title/Abstract]
21. "coronary heart diseases"[Title/Abstract]
22. "coronary stenosis"[Title/Abstract]
23. "coronary atherosclerosis"[Title/Abstract]
24. "myocardial infarction"[Title/Abstract]
25. "coronary thrombosis"[Title/Abstract]
26. "angina pectoris"[Title/Abstract]
27. 10 OR 11 OR 12 OR 13 OR 14 OR 15 OR 16 OR 17 OR 18 OR 19 OR 20 OR 21 OR 22 OR 23 OR 24 OR 25 OR 26
28. 9 AND 27

Database: Emabse

1. 'dermatomyositis'/exp
2. 'polymyositis'/exp
3. 'myositis'/exp
4. 'idiopathic inflammatory myopathy'/exp
5. 'dermatomyositis':ab,ti
6. 'polymyositis':ab,ti

7. 'myositis':ab,ti
8. 'idiopathic inflammatory myopathies':ab,ti
9. 'idiopathic inflammatory myopathy':ab,ti
10. 1 OR 2 OR 3 OR 4 OR 5 OR 6 OR 7 OR 8 OR 9
11. 'coronary artery disease'/exp
12. 'coronary disease'/exp
13. 'coronary heart disease'/exp
14. 'coronary stenosis'/exp
15. 'coronary atherosclerosis'/exp
16. 'myocardial infarction'/exp
17. 'coronary thrombosis'/exp
18. 'angina pectoris'/exp
19. 'coronary disease':ab,ti
20. 'coronary diseases':ab,ti
21. 'coronary artery disease':ab,ti
22. 'coronary artery diseases':ab,ti
23. 'coronary heart disease':ab,ti
24. 'coronary heart diseases':ab,ti
25. 'coronary stenosis':ab,ti
26. 'coronary atherosclerosis':ab,ti
27. 'myocardial infarction':ab,ti
28. 'coronary thrombosis':ab,ti
29. 'angina pectoris':ab,ti
30. 11 OR 12 OR 13 OR 14 OR 15 OR 16 OR 17 OR 18 OR 19 OR 20 OR 21 OR 22 OR 23 OR 24 OR 25 OR 26 OR 27 OR 28 OR 29
31. 10 AND 30

Database: Cochrane

1. MeSH descriptor: [dermatomyositis] explode all trees
2. MeSH descriptor: [polymyositis] explode all trees
3. MeSH descriptor: [myositis] explode all trees
4. ("dermatomyositis"):ti,ab,kw
5. ("polymyositis"):ti,ab,kw
6. ("myositis"):ti,ab,kw
7. ("idiopathic inflammatory myopathies"):ti,ab,kw
8. ("idiopathic inflammatory myopathy"):ti,ab,kw
9. 1 OR 2 OR 3 OR 4 OR 5 OR 6 OR 7 OR 8
10. MeSH descriptor: [coronary disease] explode all trees
11. MeSH descriptor: [coronary artery disease] explode all trees
12. MeSH descriptor: [coronary stenosis] explode all trees
13. MeSH descriptor: [myocardial infarction] explode all trees
14. MeSH descriptor: [coronary thrombosis] explode all trees
15. MeSH descriptor: [angina pectoris] explode all trees

16. ("coronary disease"):ti,ab,kw
17. ("coronary diseases"):ti,ab,kw
18. ("coronary artery disease"):ti,ab,kw
19. ("coronary artery diseases"):ti,ab,kw
20. ("coronary heart disease"):ti,ab,kw
21. ("coronary heart diseases"):ti,ab,kw
22. ("Coronary stenosis"):ti,ab,kw
23. ("coronary atherosclerosis"):ti,ab,kw
24. ("myocardial infarction"):ti,ab,kw
25. ("coronary thrombosis"):ti,ab,kw
26. ("angina pectoris"):ti,ab,kw
27. 10 OR 11 OR 12 OR 13 OR 14 OR 15 OR 16 OR 17 OR 18 OR 19 OR 20 OR 21 OR 22 OR 23 OR 24 OR 25 OR 26
28. 9 AND 27

2. Cardiovascular risk factors

Database: Pubmed

1. "dermatomyositis"[MeSH]
2. "polymyositis"[MeSH]
3. "myositis"[MeSH]
4. "dermatomyositis"[Title/Abstract]
5. "polymyositis"[Title/Abstract]
6. "myositis"[Title/Abstract]
7. "idiopathic inflammatory myopathies"[Title/Abstract]
8. "idiopathic inflammatory myopathy"[Title/Abstract]
9. 1 OR 2 OR 3 OR 4 OR 5 OR 6 OR 7 OR 8
10. "hypertension"[MeSH]
11. "diabetes mellitus"[MeSH]
12. "dyslipidemias"[MeSH]
13. "hyperlipidemias"[MeSH]
14. "hypertension"[Title/Abstract]
15. "diabetes mellitus"[Title/Abstract]
16. "dyslipidemias"[Title/Abstract]
17. "hyperlipidemias"[Title/Abstract]
18. 10 OR 11 OR 12 OR 13 OR 14 OR 15 OR 16 OR 17
19. 9 AND 18

Database: Emabse

1. 'dermatomyositis'/exp
2. 'polymyositis'/exp
3. 'myositis'/exp

4. 'idiopathic inflammatory myopathy'/exp
5. 'dermatomyositis':ab,ti
6. 'polymyositis':ab,ti
7. 'myositis':ab,ti
8. 'idiopathic inflammatory myopathies':ab,ti
9. 'idiopathic inflammatory myopathy':ab,ti
10. 1 OR 2 OR 3 OR 4 OR 5 OR 6 OR 7 OR 8 OR 9
11. 'hypertension'/exp
12. 'diabetes mellitus'/exp
13. 'dyslipidemias'/exp
14. 'hyperlipidemias'/exp
15. 'hypertension':ab,ti
16. 'diabetes mellitus':ab,ti
17. 'dyslipidemias':ab,ti
18. 'hyperlipidemias':ab,ti
19. 11 OR 12 OR 13 OR 14 OR 15 OR 16 OR 17 OR 18
20. 10 AND 19

Database: Cochrane

1. MeSH descriptor: [dermatomyositis] explode all trees
2. MeSH descriptor: [polymyositis] explode all trees
3. MeSH descriptor: [myositis] explode all trees
4. ("dermatomyositis"):ti,ab,kw
5. ("polymyositis"):ti,ab,kw
6. ("myositis"):ti,ab,kw
7. ("idiopathic inflammatory myopathies"):ti,ab,kw
8. ("idiopathic inflammatory myopathy"):ti,ab,kw
9. 1 OR 2 OR 3 OR 4 OR 5 OR 6 OR 7 OR 8
10. MeSH descriptor: [hypertension] explode all trees
11. MeSH descriptor: [diabetes mellitus] explode all trees
12. MeSH descriptor: [dyslipidemias] explode all trees
13. MeSH descriptor: [hyperlipidemias] explode all trees
14. ("hypertension"):ti,ab,kw
15. ("diabetes mellitus"):ti,ab,kw
16. ("dyslipidemias"):ti,ab,kw
17. ("hyperlipidemias"):ti,ab,kw
18. 10 OR 11 OR 12 OR 13 OR 14 OR 15 OR 16 OR 17
19. 9 AND 18
